# Parafoveal and peripapillary vessel density in pediatric and juvenile craniopharyngioma patients

**DOI:** 10.1038/s41598-022-09391-7

**Published:** 2022-03-30

**Authors:** Ga-In Lee, Yikyung Kim, Kyung-Ah Park, Sei Yeul Oh, Doo-Sik Kong, Sang Duk Hong

**Affiliations:** 1 Department of Ophthalmology, Samsung Medical Center, Sungkyunkwan University School of Medicine, 81 Irwon-ro, Gangnam-gu, Seoul, 06351 South Korea; 2 Department of Radiology, Samsung Medical Center, Sungkyunkwan University School of Medicine, Seoul, Korea; 3 Department of Neurosurgery, Endoscopic Skull Base Surgery Clinic, Brain Tumor Center, Samsung Medical Center, Sungkyunkwan University School of Medicine, Seoul, Korea; 4 Department of Otorhinolaryngology-Head and Neck Surgery, Samsung Medical Center, Sungkyunkwan University School of Medicine, Seoul, Korea

**Keywords:** Paediatric cancer, Optic nerve diseases, CNS cancer

## Abstract

We assessed the retinal microvascular alterations detected by optical coherence tomography angiography (OCT-A) in pediatric and juvenile craniopharyngioma (CP) patients with chiasmal compression. We included 15 eyes of 15 pediatric or juvenile CP patients and 18 eyes of 18 healthy subjects. The evaluation of vessel density from the superficial retinal capillary plexus (SRCP), the deep retinal capillary plexus, and the radial peripapillary capillary (RPC) segments was obtained by OCT-A. The association between vessel density measures and functional and structural measurements was also analyzed. There were significant reductions in the nasal sector of the SRCP (*p* < 0.0001) and all sectors of the RPC segment vessel density (nasal, temporal, and superior; *p* < 0.0001, inferior; *p* = 0.0015) in CP patients postoperatively compared to the healthy subjects. The peripapillary retinal nerve fiber layer (r = 0.6602, *p* = 0.0074) and ganglion cell-inner plexiform layer thicknesses (r = 0.7532, *p* = 0.0030) were associated with RPC segment vessel density. Visual acuity (r = − 0.5517, *p* = 0.0330) and temporal visual field sensitivity loss (r = 0.5394, *p* = 0.0465) showed an association with SRCP vessel density. In pediatric and juvenile patients with CP, parafoveal and peripapillary vascular changes following chiasmal compression were observed. The changes in vascular structures were closely related to structural and functional outcomes.

Craniopharyngioma (CP) is a rare benign tumor originating from the remnants of Rathke’s pouch, with a bimodal age distribution of 5–14 years and 50–75 years^1–3^. Because of the anatomical proximity of CP to critical neurovascular structures, pediatric CP is one of the most challenging childhood brain tumors^4,5^. Although it is defined by the World Health Organization (WHO) histologically as a benign tumor (WHO grade 1), it has a potentially malignant nature^3,6,7^. The intimate anatomical association of CP with the pituitary, hypothalamus, optic chiasm, and carotid arteries, frequently predisposes patients with CP to several adverse sequelae, including endocrine dysfunction and visual impairment^3,8^. Half of the patients present with visual dysfunction before diagnosis, which remains postoperatively and might significantly affect their quality of life^3^. Survival alone does not measure the successful management of this tumor, and changes in the visual function and alterations in its related structures should be closely examined using multiple diagnostic tools. It is well known that the peripapillary retinal nerve fiber layer (pRNFL) and ganglion cell layer complex (GCC) thinning detected by optical coherence tomography (OCT) in optic chiasmal compression have characteristic patterns and high diagnostic ability to detect band atrophy^9^. The OCT device is a useful tool to assess and reflect the degree of axonal injury-induced retinal ganglion cell death and predict visual prognosis in chiasmal compressing pituitary tumors^10–13^.

Optical coherence tomography angiography (OCT-A) is a recently developed technique to resolve the retinal and peripapillary vascular layers in three dimensions, which has led to the detection of intraocular vascular changes. Previous studies using OCT-A reported significant vascular alterations in several optic neuropathies^14–18^. Intraocular vessel densities measured by OCT-A in patients with chiasmal compressing tumors also showed a reduction compared to healthy subjects^19–21^. OCT-A results have also been reported in various diseases that may occur in children, such as amblyopia^22–24^, retinopathies^25,26^, retinoblastoma^27^, and type 1 diabetes mellitus^28,29^. However, there are no reports characterizing intraocular vessel density in pediatric and juvenile tumors mainly affecting the optic chiasm. In this study, we assessed the vessel density changes in the parafoveal and peripapillary areas in pediatric and juvenile CP patients along with alterations in the OCT parameters.

## Methods

This retrospective, cross-sectional study was approved by the Institutional Review Board of Samsung Medical Center (Seoul, Republic of Korea) and conducted in accordance with the tenets of the Declaration of Helsinki. This study included pediatric and juvenile CP patients, as well as healthy subjects under the age of 18 years who visited the Department of Neurosurgery and Neuro-ophthalmology at the Samsung Medical Center from August 1, 2018, to March 31, 2020. Informed consent was waived for the patients with CP by the Institutional Review Board of Samsung Medical Center. In all patients included in this study, CP was diagnosed preoperatively by magnetic resonance imaging. All CP patients completed radiation therapy, trans-sphenoidal tumor resections, or both. The diagnosis was confirmed pathologically after tumor resection. Only one eye of each patient was selected for analysis, that with the more severe visual field (VF) defects, and data obtained later than four months after surgery were chosen for the analysis. However, if the visual acuity was less than 20/200 in the eye with worse VF defects, the other eye with better visual acuity was selected to conduct a more accurate OCT-A examination of each patient. Patients with visual acuity less than 20/200 in both eyes were excluded. All study subjects with known ocular abnormalities (high myopia and hyperopia seen as a refractive error greater than 6.0 diopters of spherical equivalent or 3.0 diopters of astigmatism, amblyopia, any retinal disease, glaucoma, or other optic neuropathy), prior ocular surgery, or systemic or demyelinating diseases were excluded. The control eyes were enrolled from healthy volunteers who had visited the clinic for ophthalmic screening examinations. Children between 4 and 17 years of age required parental or guardian informed consent and, when appropriate, child assent, before enrollment in the study.

VF testing was assessed on standard automated perimetry with the 30–2 Swedish Interactive Thresholding Algorithm (SITA) standard protocol (Humphrey 740 Visual Field Analyzer, Carl Zeiss Meditec, Inc., Dublin, CA, U.S.A) for CP patients. The reliability criteria were a ≤ 30% false-positive or false-negative rate, and fixation loss < 20%, and the mean deviation (MD) value was used for the analysis. We also ascertained the severity of VF defects in CP patients by evaluating the central nasal mean deviation (CNMD) and the central temporal mean deviation (CTMD)^30^. Since VF loss in chiasmal compression is usually more significant in the temporal hemifield, the average VF sensitivity of the temporal and nasal hemifields was calculated in the 1/Lambert (1/L) scale and analyzed. The average VF sensitivity of the temporal and nasal hemifields was calculated by averaging the values of the eight nasal points and eight temporal points, respectively, from the total deviation plot. For each calculation, the deviation in decibels (dB) from normal at each test location was converted to unlogged 1/L units by dividing the dB value by 10 and then unlogging the quotient. The average value for each calculation was performed using the unlogged 1/L values. All included CP patients and healthy subjects completed Cirrus High-Definition (HD)-OCT analysis (Carl Zeiss Meditec AG, Jena, Germany). The OCT instrument was used to examine the macula (macula cube 512 × 128 protocol) and optic disc (optic disc cube 200 × 200 protocol) scans to obtain macular ganglion cell-inner plexiform layer (GCIPL) and pRNFL measurements. A recognition algorithm detected the inner and outer borders of the pRNFL, from a 1.73-mm diameter circle extracted from the optic nerve cube scan and centered on the optic nerve head. The distance between the two lines was measured as the pRNFL thickness at particular locations around the optic nerve (superior, inferior, temporal, and nasal). Only well-focused, well-centered scans with a signal strength above 6 units were used in the analysis. The automated Ganglion Cell Analysis (GCA) algorithm distinguished the detection of the outer border of the RNFL and the inner plexiform layers (IPL) and calculated the thickness of the ganglion cell and the IPL. GCIPL thickness was measured at particular locations around the foveal center (superior, temporal, inferior, and nasal).

**Figure 1:**
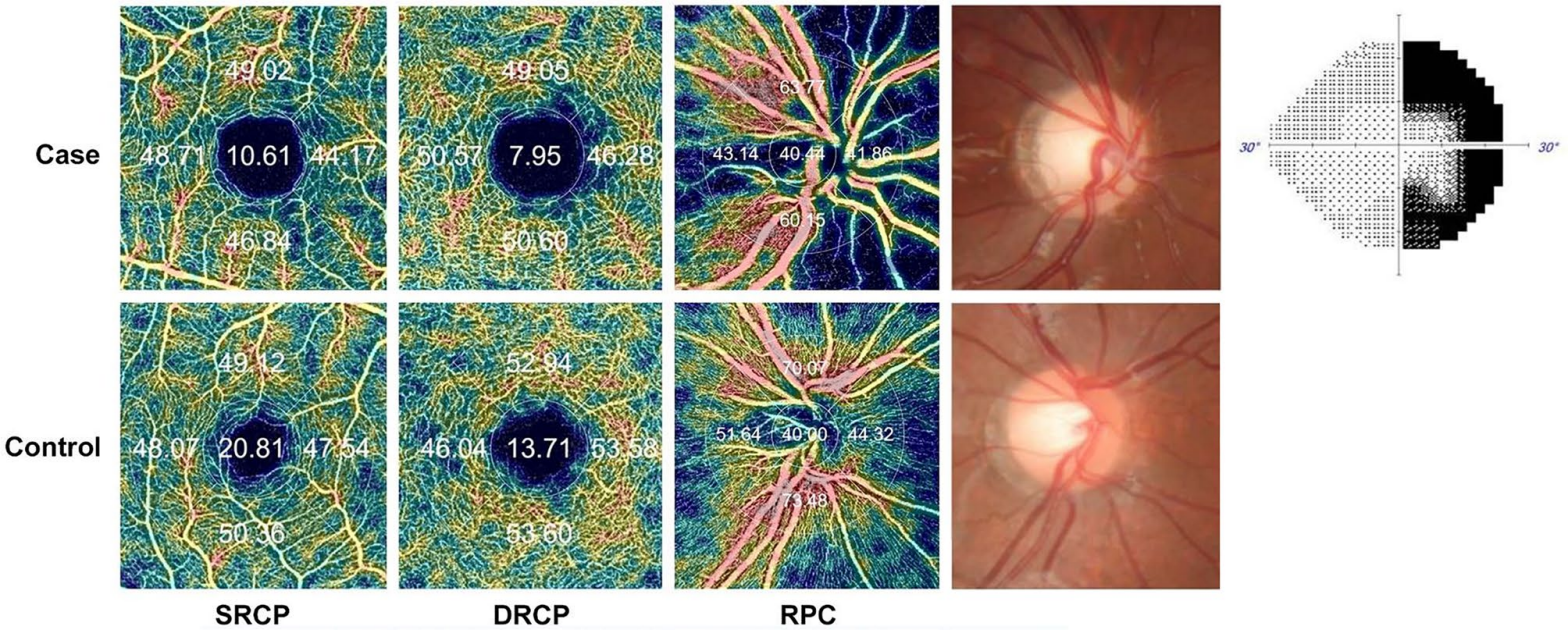
Typical parafoveal and peripapillary en-face images of eyes with pediatric and juvenile craniopharyngioma and a control eye by optical coherence tomography angiography. Color-coded flow maps reveal the automated measurement of vessel density (percentage) by auto-segmentation including the superficial retinal capillary plexus (SRCP), deep retinal capillary plexus (DRCP), and the radial peripapillary capillary (RPC) segment. The parafoveal and peripapillary vessel densities of eye with CP are lower and temporal visual field sensitivity loss is observed.

Swept-source OCT (DRI OCT Triton Plus; Topcon Corporation, Tokyo, Japan) coupled with non-invasive OCT-A technology was completed in all CP patients and healthy subjects. All OCT-A imaging was done within 4–20 months after decompression surgery. The details have been described previously^20^. The superficial retinal capillary plexus (SRCP) slab was automatically segmented from 3 µm under the internal limiting membrane (ILM) to 15 µm below the IPL, while the deep retinal capillary plexus (DRCP) slab was automatically segmented from 15 to 70 µm under the IPL, in accordance with a formerly corroborated method by Park et al.^31^. The radial peripapillary capillary (RPC) segment ranged from the ILM to the posterior boundary of the RNFL. Vessel density was determined as the percentage of the total area occupied by vessels and microvasculature, expressed as color-coded vessels quantitatively in a localized region. It was obtained by automatically applying an Early Treatment Diabetic Retinopathy Study (ETDRS) grid overlay containing the two inner rings of the ETDRS grid pattern to the fovea, which produced the density in each layer with high reproducibility and repeatability^32^. Parafoveal vessel density was determined as the mean of the four sectors in the external ring. The parafoveal ring divided the macular region into the superior, inferior, nasal, and temporal sections. The same grid was transferred to the center of the pit in the optic disc (Fig. 1). All participants completed both OCT and OCT-A imaging within one day.

The software generated TopQ image quality values for each OCT-A scan and vessel density measurement. To assess scan quality, the scan images were included based on the following quality assessment criteria suggested by Fenner et al.^33^ (1) A fine capillary network was clearly visible and easily distinguished from the background signal. Eyes with discontinuities in the vessels in the image were excluded. (2) The foveal avascular zone and disc margin lied within the central portion of the ETDRS grid applied by the OCT-A software. The location of the foveal avascular zone and optic disc margin was reviewed for accuracy and adjusted manually as needed based on the en face angiogram. (3) The RPE layer of the macular OCT scan was tilted by < 5° to the horizontal plane. (4) Less than 10% of the area of the superficial capillary plexus image contained motion artifacts, evident as horizontal white lines or black lines. (5) The RPE and individual outer segment layers were clearly visible across the horizontal length of the scan. (6) A TopQ image quality score of ≥ 60 was obtained. Expert graders reviewed and verified all images (G.I.L. and K.A.P.).

Descriptive statistics are presented as the mean and standard deviation (SD). The best-corrected visual acuity (BCVA) is expressed as a logarithm of the minimum angle of resolution (log MAR) scale. The distribution of the numerical variables was assessed using Shapiro–Wilk test for normality. The Student’s t-test was used for the group comparison of normally distributed variables. Otherwise, the Wilcoxon rank-sum test was used. The Chi-squared test was employed to compare categorical variables including gender between the groups.

Linear regression analysis was used to investigate the comparison of vessel density between two groups, following adjustments for age and spherical equivalent refractive errors (SER)^34^. The 95% confidence interval (CI) was used to determine statistical significance. Corrected *p*-values were calculated after Bonferroni’s correction for multiple comparisons.

Pearson’s or Spearman’s correlation coefficients were used to investigate the associations between vascular and structural measurements and clinical factors such as disease duration, VF sensitivity loss in the temporal and nasal hemifield, and the BCVA. Pearson’s and Spearman’s association analyses were determined by the normality test. Pearson’s analysis was used for *p*-values of > 0.05, and Spearman’s analysis was used for *p*-values of < 0.05. All statistical analyses were performed using R 3.5.1 (Vienna, Austria; http://www.R-project.org/). *P*-values (type I error) of less than 0.05 were considered statistically significant for all analyses.

## Results

This study included a total of 15 eyes of 15 patients with CP and 18 eyes of 18 healthy controls. The historical diagnoses were adamantinomatous (WHO Grade 1) type in 14 patients and papillary (WHO Grade 1) type in one patient. There were no significant differences in SER and gender between CP and healthy eyes (Table 1). The BCVA was significantly worse in the patients than in the healthy control group (*p* = 0.0031). The average VF sensitivity of the temporal and nasal hemifields (CTMD and CNMD) calculated in the 1/L scale was 670.51 ± 561.64 and 1262.23 ± 333.88, respectively. Among 15 patients, one patient had reduced visual acuity and 12 patients had VF defects with preserved visual acuity. The thickness of the pRNFL (temporal, superior, and inferior, *p* < 0.0001; nasal, *p* = 0.0060) and the GCIPL (nasal, superior, and inferior, *p* < 0.0001, temporal; *p* = 0.0010) was significantly reduced in the patient group compared to the healthy control group, including the four sectors and average values (Table 2).

**Table 1:** Characteristics of pediatric and juvenile craniopharyngioma patients and healthy controls.

Variables	Patients (n = 15)	Healthy controls (n = 18)	*p*-value
	Mean ± SD	Mean ± SD	
Gender, male/female	8/7	10/8	0.8984*
Age, years	13 ± 4	11 ± 2	0.0529^†^
Disease duration (months) [min, max]	24.63 ± 39.87 [4.10, 138.97]		
Spherical equivalent, diopters	− 1.81 ± 1.82	− 2.07 ± 1.94	0.8418^†^
Last BCVA, logMAR	0.09 ± 0.18	0.01 ± 0.02	0.0031^†^
Last VF^‡^, MD (dB) [min, max]	− 7.67 ± 6.11 [− 18.28, − 1.83]	NA	
**VF sensitivity loss**			
CTMD (1/Lambert)	670.51 ± 561.64	NA	
CNMD (1/Lambert)	1262.23 ± 333.88	NA	

*BCVA* best corrected visual acuity, *MD* mean deviation, *NA* not applicable, *SD* standard deviation, *VF* visual field, *CTMD* central temporal mean deviation, *CNMD* central nasal mean deviation.

*Chi-squared test.

^†^Wilcoxon rank-sum test.

^‡^Humphrey Field Analyzer using the 30–2 SITA-standard protocol.

**Table 2:** Comparison of retinal layer thicknesses in healthy controls and patients with pediatric and juvenile craniopharyngioma.

Variables	Patients (n = 15)	Healthy controls (n = 18)	Estimate (SE)	95% CI	Adjusted* p*-value*
**pRNFL thickness (μm)**					
Average	70.75 ± 16.55	101.33 ± 6.90	− **33.26 (4.53)**	− **42.52, **− **24.00**	**< 0.0001**
Superior	91.93 ± 26.47	130.44 ± 17.46	− **41.59 (7.69)**	− **57.33, **− **25.85**	**< 0.0001**
Inferior	89.47 ± 27.00	123.72 ± 14.35	− **40.00 (7.42)**	− **55.18, **− **24.83**	**< 0.0001**
Nasal	51.53 ± 12.92	66.56 ± 8.23	− **16.03 (4.15)**	− **24.52, **− **7.54**	**0.0060**
Temporal	50.07 ± 11.51	84.61 ± 22.74	− **35.43 (7.02)**	− **49.78, **− **21.07**	**< 0.0001**
**GCIPL thickness (μm)**					
Average	64.54 ± 12.10	83.56 ± 3.87	− **21.46 (2.89)**	− **27.38, **− **15.53**	**< 0.0001**
Superior	61.77 ± 11.99	83.17 ± 4.90	− **24.47 (3.00)**	− 30.63, − 18.31	**< 0.0001**
Inferior	63.46 ± 12.98	82.17 ± 4.83	− **20.39 (3.21)**	− **26.97, **− **13.80**	**< 0.0001**
Nasal	59.73 ± 13.46	85.17 ± 5.12	− **28.08(3.56)**	− 35.38, − 20.78	**< 0.0001**
Temporal	70.96 ± 13.41	82.61 ± 4.02	− **14.23 (3.22)**	− 20.83, − 7.62	**0.0010**

*pRNFL* peripapillary retinal nerve fiber layer, *GCIPL* ganglion cell-inner plexiform layer, *SE* standard error, *CI* confidence interval.

Values are shown as mean ± standard deviation.

*P-value was estimated by linear regression analysis with adjustments for age and spherical equivalent refractive errors and Bonferroni correction magnified by 10.

Boldface indicates statistical significance.

### Parafoveal vessel density changes by OCT-A

The average SRCP vessel density of the eyes of CP patients was reduced compared to healthy eyes following adjustment for age and SER (estimate, standard error [SE] − 3.61, 0.76; 95% confidence interval [CI] − 5.16, − 2.06; *p* < 0.0001). When compared by areas, a significant difference was found in the nasal sector in patient eyes (estimate, SE − 4.86, 1.00; 95% CI − 6.90, − 2.81; *p* < 0.0001) compared to healthy control eyes. The average DRCP vessel density of the eyes of CP patients was not significantly different from that of healthy control eyes (Table 3).

**Table 3:** Comparison of superficial, deep retinal capillary plexus and radial peripapillary capillary segment vessel densities between patients with pediatric and juvenile CP and healthy controls.

	Patients (n = 15)	Healthy controls (n = 18)	Estimate (SE)	95% CI	Adjusted *p*-value*
**SRCP, % area**					
Average	46.61 ± 2.65	49.76 ± 1.32	− **3.61 (0.76)**	− **5.16, **− **2.06**	**< 0.0001**
Superior	47.31 ± 4.27	50.15 ± 3.20	− 3.95 (1.37)	− 6.74, − 1.15	0.1080
Inferior	46.87 ± 5.16	50.37 ± 3.01	− 3.76 (1.57)	− 6.99, − 0.54	0.3525
Nasal	45.05 ± 3.43	49.30 ± 1.84	− **4.86 (1.00)**	− **6.90, **− **2.81**	** < 0.0001**
Temporal	47.20 ± 4.32	49.21 ± 2.41	− 1.88 (1.33)	− 4.60, 0.84	1.0000
**DRCP, % area**					
Average	50.86 ± 2.35	51.97 ± 2.39	− 0.99 (0.92)	− 2.88, 0.89	1.0000
Superior	52.32 ± 3.72	53.94 ± 5.66	− 1.85 (1.91)	− 5.75, 2.06	1.0000
Inferior	52.21 ± 3.81	53.46 ± 4.66	− 0.78 (1.67)	− 4.20, 2.63	1.0000
Nasal	49.26 ± 2.82	50.27 ± 2.68	− 0.80 (1.08)	− 3.01, 1.40	1.0000
Temporal	49.63 ± 4.67	50.19 ± 3.93	− 0.53 (1.53)	− 3.67, 2.61	1.0000
**RPC, % area**					
Average	52.32 ± 6.05	61.54 ± 2.38	− **10.54 (1.61)**	− **13.83, **− **7.26**	**< 0.0001**
Superior	64.19 ± 6.94	71.61 ± 2.82	− **8.69 (1.92)**	− **12.62, **− **4.76**	**< 0.0001**
Inferior	63.53 ± 8.57	72.42 ± 3.50	− **10.58 (2.36)**	− **15.40, **− **5.76**	**0.0015**
Nasal	41.48 ± 6.51	50.50 ± 3.47	− **9.87 (1.99)**	− **13.94, **− **5.80**	**< 0.0001**
Temporal	40.27 ± 6.40	50.90 ± 4.64	− **12.46 (1.89)**	− **16.32, **− **8.60**	**< 0.0001**

*SRCP* superficial retinal capillary plexus, *DRCP* deep retinal capillary plexus, *RPC* radial peripapillary capillary, *SE* standard error, *CI* confidence interval.

Values are shown as mean ± standard deviation.

*P-values were estimated by linear regression analysis with adjustment for age and spherical equivalent refractive errors and Bonferroni correction magnified by 15.

Boldface indicates statistical significance.

### Peripapillary vessel density changes by OCT-A

When peripapillary vessel density alterations were investigated, the average RPC segment vessel density in the CP eyes was significantly lower compared to the healthy control eyes following adjustment for age and SER (estimate, SE − 10.54, 1.61; 95% CI − 13.83, − 7.26; *p* < 0.0001). When compared by areas, all four sectors of the RPC segment vessel density in the eyes with CP was significantly reduced compared to the healthy control eyes (estimate, SE: superior − 8.69, 1.92; inferior, − 10.58, 2.36; nasal, − 9.87, 1.99; and temporal, − 12.46, 1.89) (Table 3).

### Association between vessel density and pRNFL thickness, GCIPL thickness, and VF sensitivity loss

**Figure 2:**
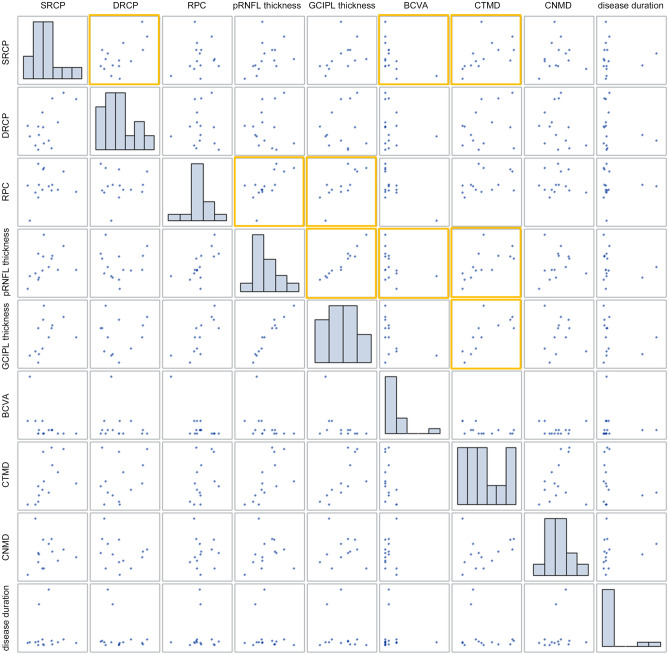
The scatter plot matrix shows the association between postoperative vessel densities and other parameters including the peripapillary retinal nerve fiber layer thickness (pRNFL), the ganglion cell-inner plexiform layer (GCIPL) thickness, logMAR best-corrected visual acuity (BCVA), visual field (VF) sensitivity, and disease duration. The pRNFL (r = 0.6602, *p* = 0.0074) and GCIPL (r = 0.7532, *p* = 0.0030) thicknesses showed significant correlations with the vessel densities in the RPC segment. Additionally, logMAR BCVA (r = − 0.5517, *p* = 0.0330) and temporal VF sensitivity loss (r = 0.5394, *p* = 0.0465) showed a significant correlation with SRCP vessel density. Otherwise, there was no significant correlation between vessel densities in the DRCP area and the inner retinal layer thicknesses or functional parameters, including VF sensitivity and BCVA.

The relationship between vessel density and other parameters, including pRNFL thickness, GCIPL thickness, logMAR BCVA, and VF sensitivity, was analyzed in patients with pediatric and juvenile CP. The pRNFL (r = 0.6602 [moderate], *p* = 0.0074) and GCIPL thicknesses (r = 0.7532 [strong], *p* = 0.0030) showed significant associations with vessel density in the RPC segment. In addition, the logMAR BCVA (r = − 0.5517, *p* = 0.0330) showed a significant moderate negative association, and temporal VF sensitivity showed a significant moderate positive association (r = 0.5394, *p* = 0.0465), with SRCP vessel density. Otherwise, there were no significant associations between DRCP vessel density and the inner-retinal layers or functional parameters, including VF sensitivity and BCVA (Table 4, Fig. 2).

**Table 4:** Correlation analysis between vessel density changes and structural, and other demographics changes.

	SRCP		RPC	
	Correlation coefficient	*p-*value	Correlation coefficient	*p-*value
Disease duration	0.1036	0.7134*	− 0.0464	0.8695*
BCVA, logMAR	− **0.5517**	0.0330*	− 0.4582	0.0859*
**VF sensitivity**				
CTMD	**0.5394**	**0.0465**^†^	0.3845	0.1747^†^
CNMD	0.0660	0.8227^†^	− 0.0388	0.8952^†^
pRNFL thickness	0.3842	0.1574^†^	**0.6602**	**0.0074**^†^
GCIPL thickness	0.4298	0.1427^†^	**0.7532**	**0.0030**^†^

*BCVA* best corrected visual acuity, *CTMD* central temporal mean deviation, *CNMD* central nasal mean deviation, *GCIPL* ganglion cell-inner plexiform layer, *pRNFL* peripapillary retinal nerve fiber layer, *RPC* radial peripapillary capillary, *SRCP* superficial retinal capillary plexus, *VF* visual field.

*P*-values by Spearman* or Pearson’s^†^ correlation with adjustment for age and spherical equivalent refractive errors.

## Discussion

This study first demonstrated alterations in the retina and peripapillary vessel density in pediatric and juvenile CP patients. The vessel density in the SRCP and RPC segment in pediatric and juvenile CP patients was significantly lower compared to the healthy control group. In addition, RPC segment vessel density was moderately related to pRNFL thickness and strongly associated with GCIPL thickness. Vessel density in the SRCP was moderately associated with BCVA and temporal VF sensitivity.

The prognosis of patients with pediatric CP is poor compared to the prognosis for adult CP patients. A 20-year population-based study in Hong Kong reported visual outcomes in 28 patients with pediatric and juvenile CP^35^. Among the 25 survivors, 15 (60%) had chronic visual impairments, including reduced visual acuity in 12 and VF defects with preserved visual acuity in three patients. In our single-institute study, 13 (87%) patients had chronic visual impairments, including reduced visual acuity in one and VF defects with preserved visual acuity in 12 patients. Almost all pediatric and juvenile CPs were reported to be the adamantinomatous type with a *CTNNB1* mutation^36,37^. In contrast, adult CPs were reported to be the papillary type^38–40^. In our study, all but one patient had adamantinomatous type CP. Specifically, adamantinomatous type CP often generates inflammatory large cystic components with rapid growth, which can injure or exert a mass effect on critical adjacent structures, necessitating urgent surgical intervention to preserve the functional structure and reduce mortality^41^. Consequently, the visual sensory structure could be more severely impaired in pediatric CP than in adult-onset CP. In 1989, Repka et al. reported the surgical outcomes of 12 children with pediatric and juvenile CP^42^. None of the children experienced an improvement in visual acuity after treatment and only one had an improvement in VF defects. During an average of 2.8 years of follow-up, there was no long-term improvement in visual acuity or VF performance in patients who presented with visual deficits after the first postoperative month^42^. Wan et al. also reported the long-term visual outcomes of pediatric and juvenile CP patients^43^. In a median follow-up of 5 years, 58% of the patients had visual impairment in at least one eye^43^. In addition to the difference in the histologic subtypes of CPs between children and adults, pediatric patients are known to present with a late diagnosis of visual symptoms^44^. In adults, more subtle changes can be detected compared to pediatric patients^44^. This might be partly related to the decreased sensitivity of field testing and poor cooperation of children compared to adults^44,45^.

Numerous OCT studies of adult-onset chiasmal compressing tumors have demonstrated sustained inner-retinal layer thinning after decompression surgery^9,13,30,46^. In chiasmal compression, the thinning of the RNFL and ganglion cell layer is prominently detected in the nasal and temporal quadrants along the horizontal meridian, forming band atrophy^13^. RNFL and ganglion cell layer thinning is attributed to retrograde axonal degeneration in patients with chiasmal compression^47^. In this study of pediatric patients, the pRNFL and GCIPL thicknesses were significantly lower along with VF defects sustained after decompression surgery, consistent with the findings of previous CP studies^30,46,48^.

Recently, several studies reported the OCT-A results in chiasmal compression in adult patients^19–21^. Suzuki et al. reported that eyes with temporal hemianopsia, referred to as band atrophy, showed a significant reduction in vessel density in the circumpapillary and macular areas^19^. Dallorto et al. reported a significant reduction in peripapillary and superficial macular vessel density in adult patients with pituitary adenomas^21^. Our previous study also reported similar results that peripapillary and superficial parafoveal vessel density in adult patients with chiasmal-compressing pituitary tumors was significantly lower compared to healthy controls^20^. In this study of pediatric and juvenile patients, the superficial parafoveal and peripapillary vessel densities were significantly reduced compared to healthy controls. Our study confirmed that intraocular vessel density was also significantly altered in pediatric and juvenile patients with chiasmal compression.

In the association analysis of adult-onset pituitary tumors, meaningful associations between vessel density measurements by OCT-A and OCT parameters or functional parameters were demonstrated in previous studies^19–21^. Suzuki et al. reported that peripapillary and macular vessel density correlated strongly with pRNFL and GCC thickness and the degree of VF sensitivity loss^19^. Dallorto et al. reported that peripapillary vessel density was significantly correlated with pRNFL thickness and VF defects^21^. This study on pediatric and juvenile CP also showed a significant association between GCIPL and pRNFL thicknesses and intraretinal vascular changes. More thinning of the GCIPL and pRNFL was associated with more reductions in intraretinal vessel density. There was also a moderate, but significant association between superficial parafoveal vessel density and visual acuity and temporal VF sensitivity in this study. These results suggest that the retinal vessel density status reflects the degree of remaining central vision and visual fields in pediatric and juvenile CP patients. Our results demonstrated that intraocular vascular alterations measured by OCT-A reflected the pathological changes and functional deficits of chiasmal compression in pediatric and juvenile populations similar to adult patients with chiasmal compressing lesions^19–21^. Our study has the strong point that we homogeneously included pediatric and juvenile CP patients. Unlike pituitary adenomas, which were mainly included in other previous studies where adults were the main subjects^19,20^, CP has anatomical continuity with critical neurovascular structures, leading to severe adverse sequelae including visual impairment after decompression surgery. Thus, due to the distinct nature of CP, analyzing CP patients separately from other types of chiasmal compressing tumors may have clinical significance^49,50^.

There were several possible limitations to this study. First, our study population was relatively small and the patients were inconsistently followed for 4–20 months after decompression surgery, in a retrospective manner. A larger and longer-term cohort study is required to investigate the long-term visual function outcomes and predictive factors. Second, vessel density measurements by OCT-A use the motion contrast created by flowing erythrocytes, but several factors encompass the measurement of blood flow quantitatively. We could not exclude the probability of hidden micro-swelling of the optic nerve or slight axonal edema affecting the assessment of changed vascular density.

## Conclusion

Despite these limitations, the study first demonstrated the changes in retinal microvasculature in pediatric and juvenile CP patients. Eyes with pediatric and juvenile CP showed a diminution in parafoveal and peripapillary vessel density along with thinning of the pRNFL and GCIPL thicknesses. The significant association between intraretinal microvasculature and visual acuity and temporal VF sensitivity in this study suggests a potential indirect role of OCT-A measurements in the assessment of visual function in pediatric patients with brain tumors. Further studies are required to confirm our findings and reveal the proper clinical implications of these intraocular vascular alterations in pediatric and juvenile CP patients.
